# Intracranial Arterial Calcification and Intracranial Atherosclerosis: Close but Different

**DOI:** 10.3389/fneur.2022.799429

**Published:** 2022-02-08

**Authors:** Heng Du, Jia Li, Wenjie Yang, Daniel Bos, Lu Zheng, Lawrence Ka Sing Wong, Thomas W. Leung, Xiangyan Chen

**Affiliations:** ^1^Department of Health Technology and Informatics, The Hong Kong Polytechnic University, Kowloon, Hong Kong SAR, China; ^2^Department of Critical Care Medicine, Ruijin Hospital, Shanghai Jiao Tong University School of Medicine, Shanghai, China; ^3^Department of Diagnostic Radiology and Nuclear Medicine, University of Maryland School of Medicine, Baltimore, MD, United States; ^4^Department of Radiology and Nuclear Medicine, Department of Epidemiology, Erasmus MC University Medical Center, Rotterdam, Netherlands; ^5^Department of Clinical Epidemiology, Harvard TH Chan School of Public Health Boston, Cambridge, MA, United States; ^6^Department of Medicine and Therapeutics, Prince of Wales Hospital, The Chinese University of Hong Kong, Hong Kong, China

**Keywords:** intracranial arterial calcification, intracranial atherosclerotic disease, computed tomography, vessel-wall magnetic resonance imaging, plaque morphology

## Abstract

**Background and Purpose:**

Intracranial arterial calcification (IAC) may be present in the intimal or medial arterial layer. This study aimed to elucidate the link between the calcification and atherosclerotic disease in the intracranial vasculature.

**Methods:**

Consecutive patients with acute ischemic stroke were included. Bilateral intracranial segment of the internal carotid artery, M1 segment of the middle cerebral artery, intracranial segment of the vertebral artery, and the basilar artery were visualized by the multi-detector computed tomography (CT) and vessel-wall magnetic resonance imaging (vwMRI) within 14 days after stroke onset. IAC was into the intimal or medial pattern. Subsequently, on the vwMRI, we assessed the luminal stenosis, eccentricity, plaque burden, and intraplaque hemorrhage (IPH) as markers of atherosclerosis at each IAC site.

**Results:**

Among 69 patients with stroke, IAC was identified in 35% of (161/483) artery segments, of which 61.5% were predominantly intimal calcification and 38.5% were predominantly medial calcification. About 79.8% of intimal calcifications and 64.5% of medial calcifications co-existed with atherosclerotic plaques. Intimal calcification was associated with luminal stenosis (*p* = 0.003) caused by atherosclerotic lesions. Compared with the medial IAC, intimal IAC was more often accompanied by eccentric plaques (*p* = 0.02), larger plaque burden (*p* = 0.001), and IPH (*p* = 0.001).

**Conclusion:**

Our multimodal imaging-based comparison study on intracranial arteriosclerosis demonstrated that intimal IAC, compared with medial IAC, was more often accompanied by the luminal stenosis, larger plaque burden, eccentricity, and IPH, providing strong evidence for clinical evaluation on the mechanism, risk, and prognosis of ischemic stroke.

## Introduction

Intracranial arterial calcification (IAC) is commonly seen in major cerebral arteries (1–3) and established as an independent risk factor for stroke (4). In previous studies, grading by extent and thickness qualitatively (5) and calculating volume with specific software quantitatively (6) were the most frequently applied evaluation methods on IAC. However, both methods lack consideration about the inherent difference between IACs in separate layers of vessel wall, which may act as a potential cause to contradictory findings (3, 7).

Intracranial arterial calcification mainly involves the intimal and medial layer, which could vary in both histopathological feature and imaging (8). Histopathological study showed that intracranial arteries with intimal calcification had severer lumen stenosis as compared to that without intimal calcification (9, 10). Imaging based studies reported that the medial calcification was associated with poor collateral status (11) and higher frequency of post-intravenous-thrombolysis hemorrhage (12) compared with intimal calcification. The difference between IAC patterns brought about a need of advanced details about the correlation of IAC with intracranial atherosclerotic disease (ICAD). Therefore, we aimed to compare the morphological features between intracranial plaques with intimal calcification and those with medial calcification.

## Materials and Methods

### Subjects

The study was approved by the Clinical Research Ethics Committee of the Chinese University of Hong Kong. This was a retrospective study based on a prior prospective study. Patients who underwent computed tomography (CT), routine magnetic resonance imaging (MRI), magnetic resonance angiography (MRA), and vessel-wall magnetic resonance imaging (vwMRI) within 14 days after the onset of ischemic stroke or transient ischemic attack (TIA) from 2014 to 2018 at the Prince of Wales Hospital, Hong Kong were included in this study. The exclusion criteria were as follows: (1) other vasculopathies, such as dissection and vasculitis; and (2) incomplete image data or poor image quality.

### CT Protocol and Post-processing

A routine non-enhanced brain CT was performed using a 64-slice multi-detector row CT system (Light speed 64 plus, General Electric, Milwaukee, WI, USA) with acquisition parameters as follows: slice thickness 5 mm; 120 kVp, 170 mAs, and 1 s per rotation. All axial images were reconstructed at 0.625-mm intervals. Each CT scan was obtained in axial mode, with tilting along the occipital-meatal line.

**Figure 1:**
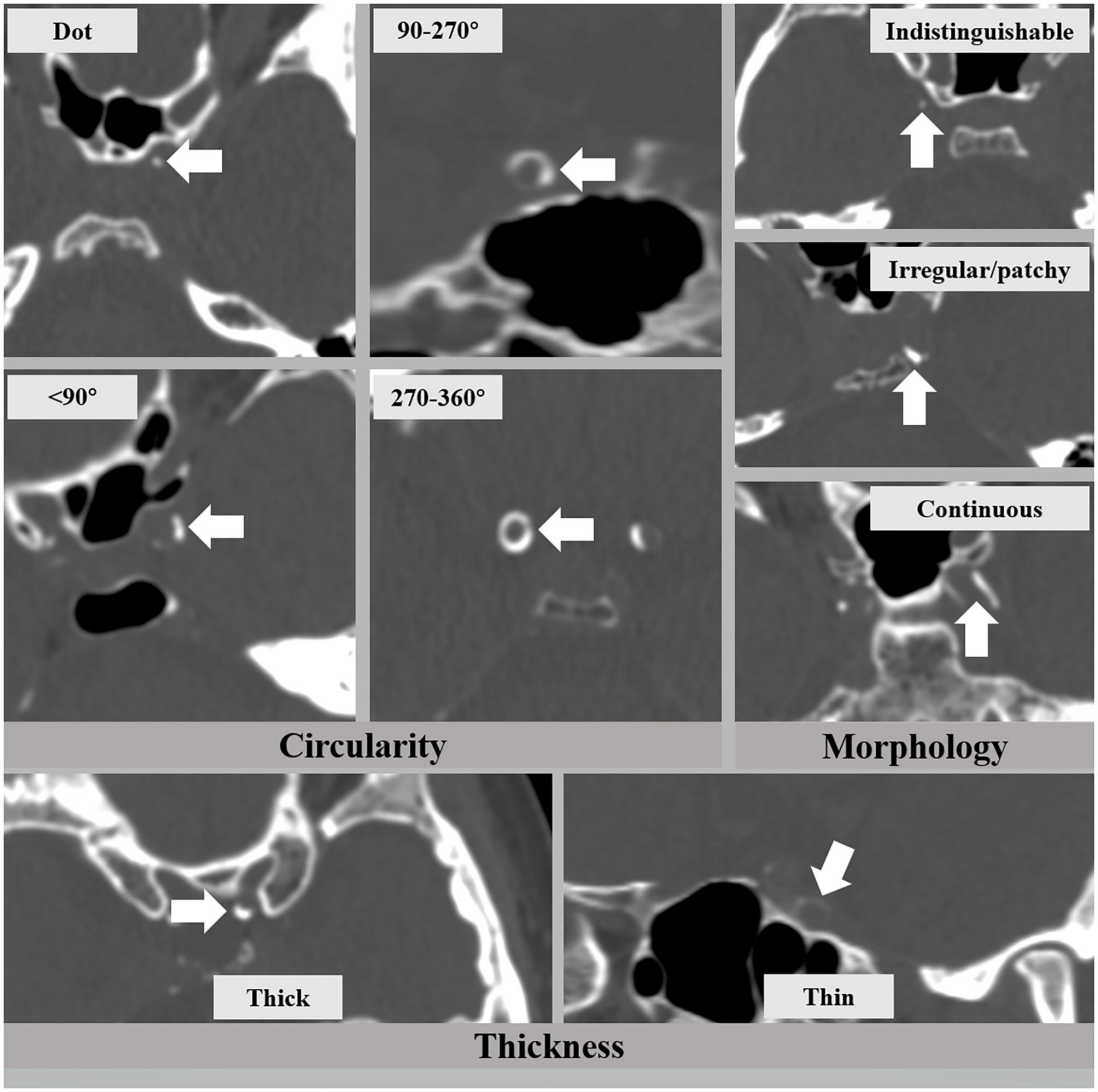
The intracranial arterial calcification (IAC) scoring model that consists of circularity (dot, <90, 90–270, and 270–360 degrees), thickness (thick ≥ 1.5 mm and thin < 1.5 mm), and morphology (indistinguishable, irregular/patchy, and continuous).

The presence and patterns of IAC were assessed on reconstructed CT images by two readers (H.D. and L.Z.) who had more than 3 years of experience in brain CT imaging and were blinded to the clinical and MRI data. Each major intracranial artery segment was assessed, including the cavernous segment (C4) of the internal carotid artery (ICA), the supraclinoid and ophthalmic segment (C5-6) of the ICA, M1 segment of the middle cerebral artery (MCA), intracranial segment (V4) of the vertebral artery (VA), and the basilar artery (BA). IAC was defined as hyperdense foci over 130 Hounsfield units (HU) (refs). The patterns of IAC were categorized into intimal or medial according to a previously established scoring model (8), in which circularity (1 for dot, 2 for <90 degrees, 3 for 90–270 degrees, and 4 for 270–360 degrees), thickness (1 for thick IAC ≥ 1.5 mm and 3 for thin IAC < 1.5 mm), and morphology along the long axis of the artery (0 for indistinguishable, 1 for irregular/patchy, and 4 for continuous) were evaluated and summed up as a total score (Figure 1). A total score that ranged from 1 to 6 indicated predominantly intimal calcification (intimal IAC) and 7–11 was deemed as predominantly medial calcification (medial IAC).

### MRI Protocol and Post-processing

Vessel-wall MRI was performed using a 3.0 T Achieva MR system (Philips Healthcare, Cleveland, OH, USA) with an 8-channel head coil. The time-of-flight MR angiography (TOF-MRA) sequence and transverse 3D T1-weighted volumetric isotropic turbo spin-echo acquisition (T1w-VISTA) sequence were acquired. Parameters for TOF-MRA were as follows: field-of-view (FOV) 200 × 200 × 56 mm^3^, acquired resolution 0.4 × 0.6 × 0.7 mm^3^, repetition time (TR)/echo time (TE) 23/3.5 ms. Parameters for T1w-VISTA: FOV 200 × 167 × 45 mm^3^, acquired resolution 0.6 × 0.6 × 1.0 mm^3^, reconstructed resolution 0.5 × 0.5 × 0.5 mm^3^, TR 1,500 ms, and TE 36 ms. vwMRI images were repeated after the injection of gadolinium-containing contrast agent (Dotarem; Guerbet, Roissy CdG Cedex, France) intravenously with a ratio of 0.2 ml/kg and an injection rate of 3.5 ml/s.

**Figure 2:**
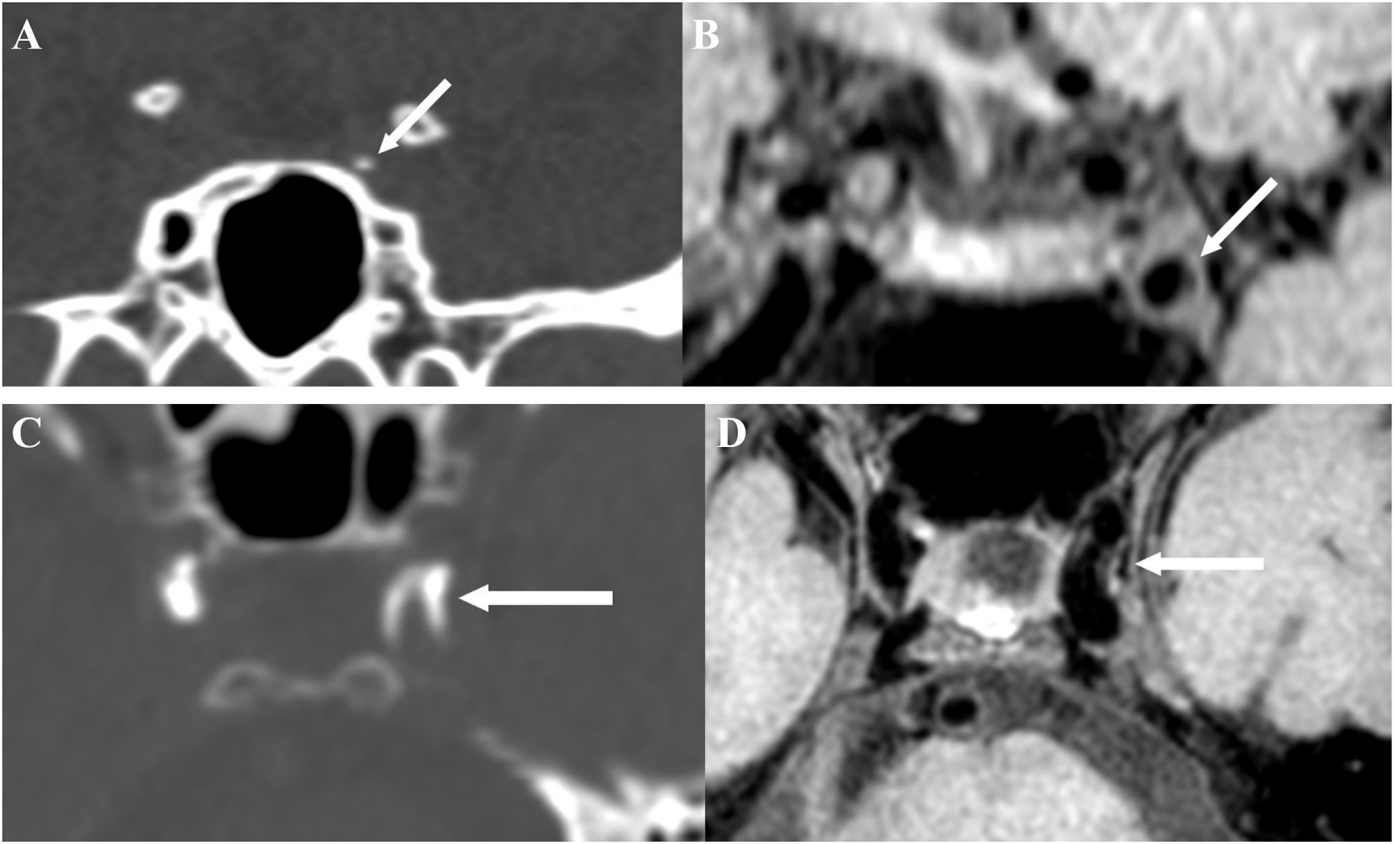
Intimal calcification detected by brain CT **(A)** and the corresponding site on vessel-wall MRI (vwMRI) **(B)**. Medial calcification detected by brain CT **(C)** and the corresponding site on vwMRI **(D)**.

**Figure 3:**
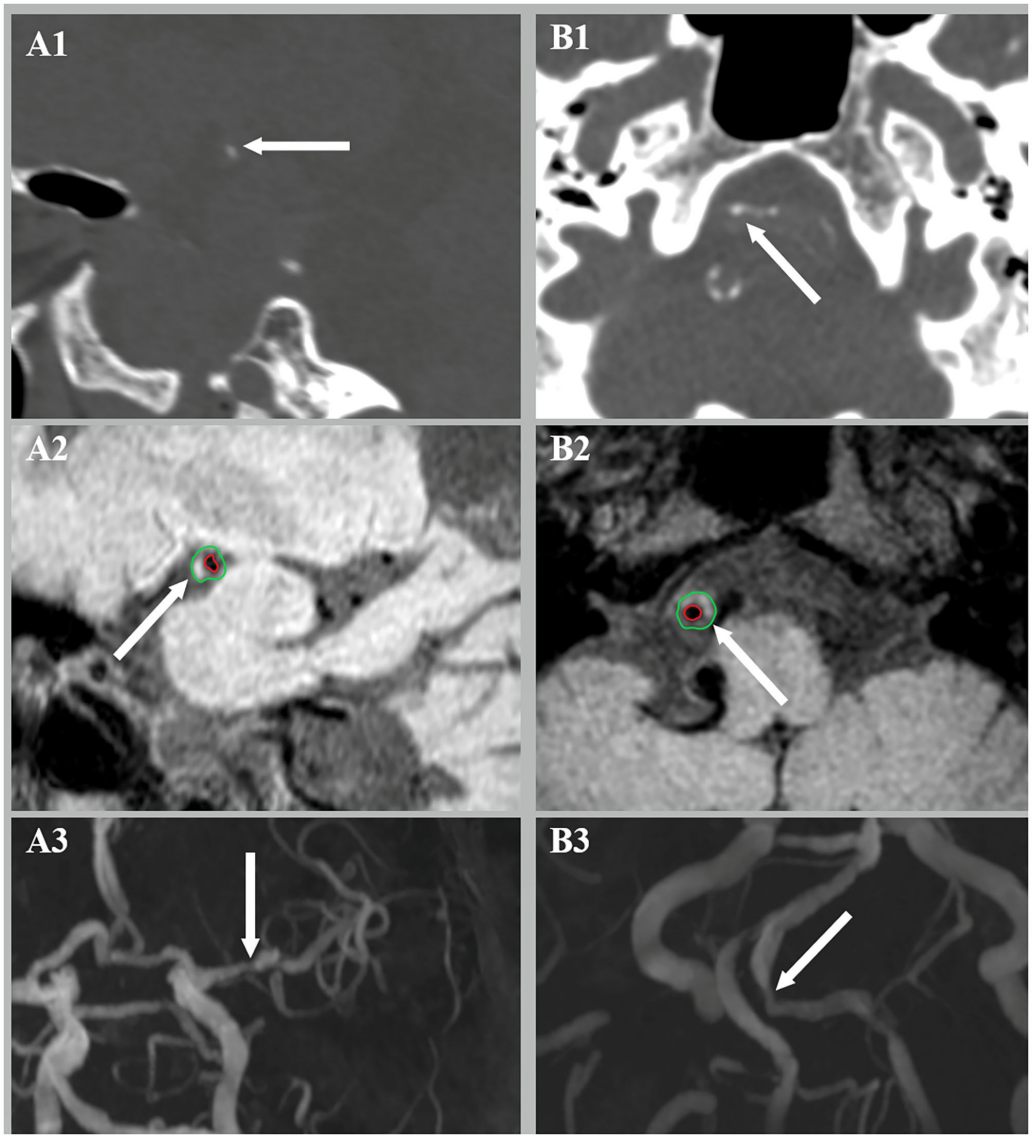
Intraplaque hemorrhage (IPH) **(A2)** and luminal stenosis **(A3)** were detected at the corresponding site of intimal calcification **(A1)**. Medial calcification **(B1)** was found with concurrent IPH **(B2)** and luminal stenosis **(B3)**.

The presence of atherosclerotic lesion was identified on reconstructed images using the OsiriX DICOM Viewer (Pixmeo SARL, Bernex, Switzerland) by two readers (H.D. and L.Z.) who were blinded to the clinical data and CT findings. Images from TOF-MRA were used for identifying luminal stenosis (Figure 2). Stenosis was evaluated on reconstructed images and maximum intensity projection (MIP) images with four degrees, such as (1) mild stenosis, (2) moderate stenosis, (3) severe stenosis, and (4) signal void. Images from vwMRI were reconstructed perpendicular to the axis of vessel by OsiriX DICOM Viewer (Pixmeo SARL, Bernex, Switzerland). An atherosclerotic plaque was defined as vessel wall thickening on pre- and post-contrast vwMRI. The outer-wall area (OWA) and lumen area (LA) were measured manually by tracing the outer interface and lumen-intimal interface, respectively (Figure 3). Plaque burden, maximum, and minimum wall thickness were derived accordingly. Plaque burden was defined as (OWA-LA)/OWA. Eccentricity index was defined as (maximum wall thickness – minimum wall thickness)/maximum wall thickness. A plaque was defined as eccentric if the index was ≥0.5 or concentric if <0.5, as described in a prior study (13). Intraplaque hemorrhage (IPH) was defined as an area of hyperintensity (>150% of the adjacent area of the vessel wall) within the plaque (Figure 3) (14).

### Imaging Matching

After the identification of IAC on CT and plaques on vwMRI, a side-by-side analysis was conducted to check whether the stenosis and plaques were at each corresponding site of IAC based on anatomy.

### Statistical Analysis

For statistical analysis, IBM SPSS (20.0, SPSS, Inc) was used. Continuous variables were expressed by mean ± SD and categorical variables were presented as numbers and percentages. *T*-tests were used for comparison of age and plaque burden between groups. Pearson's chi-square test or Fisher's exact test was used for comparisons of the distribution patterns of IAC, category of luminal stenosis, stenotic degree, eccentricity, and IPH between plaques with intimal calcification and that with medial calcification. Multivariable regression was used to evaluate the association between IAC and luminal stenosis, eccentricity, and IPH. A two-sided *p* < 0.05 was considered statistically significant.

## Results

In total, 79 patients were included after four patients were excluded due to incomplete images or poor image quality. It was found that 69 (87.3%) patients had calcification in one or more major intracranial arteries, and the other 10 patients showed no calcification. Patients with IAC were generally older than those without IAC (mean age, 65.3 ± 10.7 vs. 54.1 ± 8.7 years, *p* = 0.001).

Among patients with IAC, 44 out of 69 (63.8%) patients were men (mean age, 64.0 ± 11.2 years) and 25 were women (mean age, 67.5 ± 9.8 years). History of smoking was found in 22 out of 69 patients (31.9%). In addition, 54 out of 69 patients (78.3%) had hypertension while the prevalence of diabetes and hyperlipidemia was 39.1 and 50.7%, respectively. Among 10 patients without IAC, the number of male and female was four and six, respectively. Of the 10 patients, six patients had history of smoking, seven had hypertension, two had diabetes, and three had hyperlipidemia.

### Distribution of Intimal Calcification and Medial Calcification

Among the 69 patients with IAC, 65 (94.2%) had 1–4 calcifications and one patient had as many as 7 calcified arteries. A total of 460 segments were examined, out of which 161 segments (35.0%) with either intimal or medial calcification were identified in brain CT (Table 1). Most IACs were located in the cavernous (37.9%) and supraclinoid-to-ophthalmic (34.2%) segment of ICA, followed by the V4 segment of the VA (20.5%), M1 segment of the MCA (5.0%), and the BA (2.5%).

**Table 1:** Distributions of intracranial arterial calcification (IAC) and IAC with intracranial atherosclerotic disease (ICAD) in major intracranial artery segments.

	**Calcification**			**Calcification with ICAD**
	**Intimal**	**Medial**	**Total**	
ICA				
C4	34 (34.3%)	27 (43.5%)	61 (37.9%)	38 (31.9%)
C5-6	30 (30.3%)	25 (40.3%)	55 (34.2%)	39 (32.8%)
M1	7 (7.1%)	1 (1.6%)	8 (5.0%)	8 (6.7%)
V4	24 (24.2%)	9 (14.5%)	33 (20.5%)	30 (25.2%)
BA	4 (4.0%)	0 (0.0%)	4 (2.5%)	4 (3.4%)
Total	99	62	161	119

*ICA, internal carotid artery; C4, cavernous segment; C5-6, supraclinoid and ophthalmic segment; M1, M1 segment of middle cerebral artery; V4, V4 segment of vertebral artery; BA, basilar artery*.

**Figure 4:**
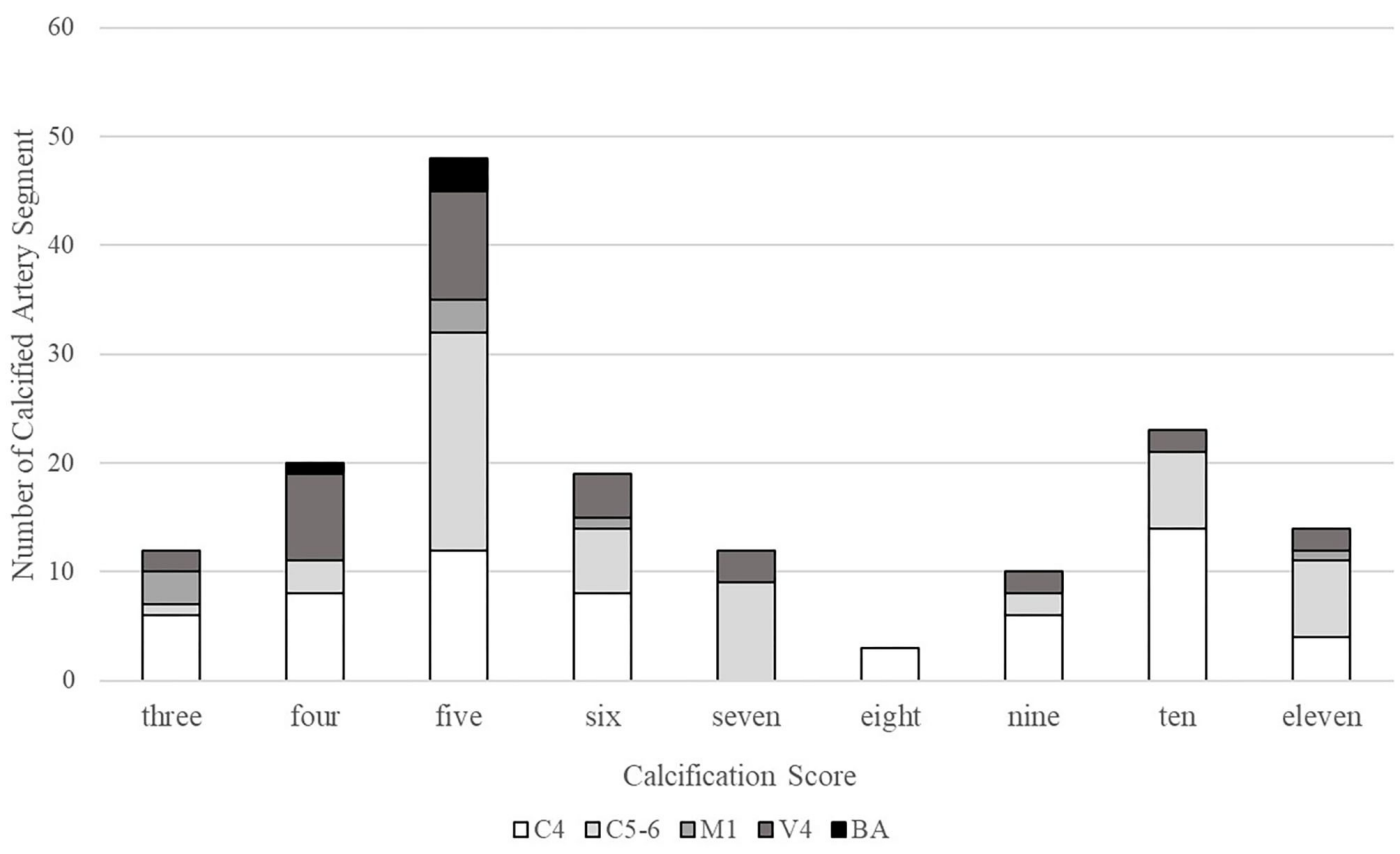
Distribution of the cumulate calcification scores. C4, cavernous segment of internal carotid artery; C5-6, supraclinoid and ophthalmic segment of internal carotid artery; M1, M1 segment of middle cerebral artery; V4, V4 segment of vertebral artery; and BA, basilar artery.

Of the 161 IACs, 99 (61.5%) were categorized as predominantly intimal IACs and the other 62 (38.5%) as predominantly medial IACs (Table 1). Figure 4 shows the distribution of the cumulate calcification score. Among intimal IACs, 48 were given a score of 5, accounting for the majority (48.5%). A score of 4 was identified in 20 intimal IACs (20.2%), followed by a score of 6 in 19 intimal IACs (19.2%) and a score of 3 in 12 intimal IACs (12.1%). Among medial IACs, a score of 10 was more prevalently identified than others (23 in 62 cases, 37.1%). The occurrences of the scores of 7 (12 in 62, 19.4%), 9 (10 in 62, 16.1%), and 11 (14 in 62, 22.6%) were close. Only 3 cases were given a score of 8.

Intimal IAC was frequently detected in the cavernous (34.3%) and supraclinoid-to-ophthalmic (30.3%) segments of ICA and the V4 segment of the VA (24.2%), while medial IAC was more prevalently present in the cavernous (43.5%) and supraclinoid-to-ophthalmic (40.3%) segment of the ICA. Compared with intimal IACs, medial IACs were more predominantly distributed in the C4–C6 segment of the ICA (64.6 vs. 83.9%, *p* = 0.008) and less frequently involved the intracranial VA (*p* = 0.137).

### Imaging Characteristics of ICAD With Intimal or Medial Calcification

Corresponding intracranial artery segments from 10 patients without IAC were included as control. A total of 70 calcium-free segments were examined, of which 40 (57.1%) were identified as “non-calcified plaques”.

Comparing atherosclerotic lesions assessed by vwMRI, 79.8% (79/99) co-existed with atherosclerotic plaque while 64.5% (40/62) had concurrent plaque. Luminal stenosis was more frequently found in plaques with intimal IAC than plaques with medial IAC (74.7 vs. 47.5%; *p* = 0.003) and non-calcified plaques (74.7 vs. 45.0%; *p* = 0.001) (Table 2). There was no significant difference in the category of stenosis (“mild and moderate stenosis” vs. “severe stenosis and signal void”) among plaques with intimal IAC, plaques with medial IAC, and non-calcified plaques. More plaques with intimal IAC showed as eccentric than plaques with medial IAC (67.1 vs. 45.0%; *p* = 0.02) and non-calcified plaques (67.1 vs. 42.5%; *p* = 0.01). Plaques with intimal IAC had higher plaque burden compared with plaques with medial IAC (0.718 ± 0.104 vs. 0.644 ± 0.122, *p* = 0.001) and non-calcified plaques (0.718 ± 0.104 vs. 0.656 ± 0.104, *p* = 0.003). IPH was found in 35.4% of plaques with intimal IAC, much higher than that in plaques with medial IAC (7.5%, *p* = 0.001) and non-calcified plaques (15.0%, *p* = 0.02). No difference in the presence of luminal stenosis, plaque burden, eccentric pattern, or IPH was found between plaques with medial IAC and non-calcified plaques.

**Table 2:** Vessel wall morphology of arterial segments with ICAD and concurrent intimal (or medial) calcification and arterial segments with non-calcified atherosclerotic plaques.

	**ICAD with calcification**		
**Change in vessel wall**	**ICAD with intimal calcification**	**ICAD with medial calcification**	^*^Non-calcified plaque
Total	79	40	40
**Vessel stenosis**			
**Total stenotic segments**	**59 (74.7%)**	**19 (47.5%)**	**18 (45.0%)**
**Mild-to-moderate stenosis**	**35 (44.3%)**	**14 (35.0%)**	**13 (32.5%)**
Mild	22 (27.8%)	8 (20.0%)	9 (22.5%)
Moderate	13 (16.5%)	6 (15.0%)	4 (10.0%)
**Severe stenosis and signal void**	**24 (30.4%)**	**5 (12.5%)**	**5 (12.5%)**
Severe	12 (15.2%)	3 (7.5%)	3 (7.5%)
Signal void	12 (15.2%)	2 (5.0%)	2 (5.0%)
**Plaque burden**	0.718 ± 0.104	0.644 ± 0.122	0.656 ± 0.104
**Plaque morphology**			
Concentric plaque	26 (32.9%)	22 (55.0%)	23 (57.5%)
Eccentric plaque	53 (67.1%)	18 (45.0%)	17 (42.5%)
**Intraplaque hemorrhage**	28 (35.4%)	3 (7.5%)	6 (15.0%)

* *Non-calcified plaques were identified via vessel-wall MRI (vwMRI) among 70 major artery segments from 10 patients without intracranial arterial calcifications on brain CT*.

The multivariable regression model revealed that the presence of luminal stenosis (*OR* 2.140; 95% *CI*, 1.001–4.578; *p* = 0.05), eccentric plaque (*OR* 2.621; 95% *CI*, 1.376–4.993; *p* = 0.003), and IPH (*OR* 3.269; 95% *CI*, 1.376–7.767; *p* = 0.007) were associated with intimal IAC, after adjusting for age, gender, hypertension, diabetes, hyperlipidemia, and smoking.

## Discussion

In the present study, we found that medial calcifications were prevalently distributed in the intracranial ICA while the distribution of intimal calcifications was more equal. Compared with plaques coexisting with medial IAC and non-calcified plaques, plaques with intimal IAC were more associated with the presence of luminal stenosis, eccentric plaques, higher plaque burden, and the presence of IPH.

A prior CT study reported that the intracranial ICA was the most frequently affected segment by IAC (60%), whereas the MCA and the BA were rarely affected (5%, respectively) (15). A possible reason of the high prevalence of IAC around the cavernous and carotid siphon may be the tortuous anatomical configuration, which may result in abnormal hemodynamics (16). In the present study, medial calcifications were found predominantly distributed in the intracranial segment of the ICA and the VA, consistent with previous histopathological findings (9, 10). It is noteworthy that a prior autopsy study reported an extremely high prevalence of calcification (97.4%) along the medial layer in the intracranial ICAs (9) and a relatively high prevalence of medial calcification (36.8%) among the intracranial VAs (10). In the present study, such high prevalence was not found in either the ICA or the VA. As reported in the autopsy, 36% of the medial calcifications had calcified circumference <50% (9). With less circumference affected, medial IAC may have cluster-like shape instead of circular, which may be categorized as intimal calcification on CT.

Histopathological findings demonstrated that an intimal IAC was present prevalently in the advanced stage of atherosclerosis (10). Being granules initially, intimal calcification will grow in size with constant fusion and will turn into large clusters (17). Previous autopsy study revealed a marked concurrence of atherosclerotic lesion among intimal calcifications (85%) but not vice-versa (62%) in the carotid artery (9), partly matching the concurrence of medial calcification and intracranial atherosclerosis (64.5%) in the present study. Although medial IAC is thought to form independently of the presence of atherosclerosis, concurrent intracranial plaques were in 38% of medial IACs on histology (10). Luminal stenosis, prevalently presented in ICAD and resulting in multiple stroke patterns (18), is one of the most critical causes of severe stroke and recurrent strokes (19, 20). In the present study, intimal IAC was found independently associated with the presence of luminal stenosis. However, no significant difference in stenotic severity was identified between atherosclerotic lesions with intimal or medial IACs. Previous histological findings suggested that among major cerebral arteries, the presence of intimal IAC was associated with severer luminal stenosis (10). Since the medial IACs in the comparison of our study were concurrent with atherosclerosis while the referred histological study included all medial IACs (with or without atherosclerosis) (10), the prevalence of narrowed artery might be increased in our study.

With luminal stenosis exceeding over 70%, eccentric plaque in the extracranial carotid artery becomes associated with a significant increased incidence of ipsilateral stroke (21). Similar findings were found in intracranial arteries. Most of the progressive ICAD presented eccentric plaques (84.4%) (22), which was much more prevalent than other kinds of vasculopathy (23). However, discrepancies exist. First, the prevalence of eccentric plaque differs between the anterior circulation and posterior circulation (24). Second, eccentricity was reported either associated (25) or not (26) with stroke according to different studies. Furthermore, a histological study demonstrated a similar proportion of eccentric and concentric lesions among ICAD of general adults (27). One of the causes might be the inclusion criteria: progressive ICAD is always accompanied by luminal stenosis (23) while recruited samples from general adults(27) may consist of lesions from different stages of ICAD. The process of change in histological feature during the evolvement of atherosclerosis can explain some of the discrepancies and more studies upon eccentricity are required to clarify its relevance to ICAD and strokes. Except for eccentricity, plaque burden is another notable factor that tends to be underestimated by the traditional lumen angiographic imaging as a result of vessel wall enlargement induced by positive vascular remodeling during the progress of ICAD (25). Previous study reported the association of plaque burden with the National Institutes of Health Stroke Scale (NIHSS) scores (28), indicating a latent impact of plaque burden on stroke severity. Additionally, a recent vwMRI study revealed that plaque burden was significantly correlated to recurrent stroke with every 10% increase in plaque burden leading to a 2.26-fold higher risk of stroke recurrence (29). By connecting IAC with eccentricity and plaque burden, the present study was thought to provide an improvement in assessing the risk and prognosis of ischemic stroke from the other side.

Intraplaque hemorrhage, attributed to immature and fragile neovessels (30), is an important marker of plaque vulnerability and predictor of ischemic events (31–33). By a sudden expansion in plaque components, IPH can exacerbate luminal stenosis, or worse, result in plaque rupture, leading to *in situ* thrombosis and distal embolism (34). In prior studies, correlation of IAC and plaque vulnerability was controversial (3, 7). A latent reason for such controversy could be the unclassified IAC patterns. The present study provided additional evidence by identifying intimal calcification as an independent risk factor for IPH. In calcified coronary arteries with plaque rupture, the junctions between superficial calcified nodules and soft plaque were vulnerable to shear stress that may cause plaque rupture (35). Similar findings were reported in terms of carotid arteries (36). Since intimal calcifications frequently exist in the superficial layer of the intracranial vessel wall, we deduce that the possible mechanism of IAC-induced IPH could be the deformation and rupture of intraplaque neovessels resulting from the increased shear stress upon the plaque surface. To test this hypothesis, further studies on the mechanical conditions and hemodynamics of IAC might be needed.

The organization of intracranial arteries have unique features compared with other vessels: a thinner muscular layer, a less abundant adventitia and a hypoplastic external elastic membrane (37). These features make intracranial arteries frailer under external pressure than other vessels. During mechanical endovascular thrombectomy or arterial stenting, one concern is arterial calcification. The presence of calcification is associated with arterial stiffness (38), which is thought to make vessel wall more brittle during endovascular procedures. As a result, proper surgical instruments, delicate control of balloon dilatation during stenting (or thrombectomy) will significantly lower the risk of unexpected vessel rupture. Another concern could be the smoothness of stent retrieving during endovascular thrombectomy. Large-calcified lesions or stiffened vessel wall may block the extraction of thrombus and therefore increase the passes of retriever, leading to prolonged procedure time and worse outcome (39). According to our study, intimal IAC more often coexists with luminal stenosis, larger plaque burden, eccentric plaque, and IPH. By distinguishing the pattern of IAC, we aim to provide evidence for decision making in pre- and post-treatment assessment. Future studies will be needed to study the latent impacts of IAC pattern on the pathophysiological process of stroke.

This study had several limitations. First, the sample size was not large enough to compare vascular risk factors of IAC patterns. Second, all patients recruited were diagnosed with either infarction or TIA, of whom the prevalence of IAC and progressive atherosclerosis could be higher than that among general adults. Third, it is still difficult to link IAC to territorial stroke by the findings in our study. Except for ICAD in the carotid artery, MCA lesions (either luminal stenosis or non-stenotic plaques) also takes a dominant role in the ischemic events of anterior circulation. Under the coexistence of multiple atherosclerotic lesions in both ICA and MCA, it is difficult to identify the culprit lesion that accounts for infarction (or TIA), especially artery-to-artery embolism and hypoperfusion. On the other hand, as single subcortical infarction is prevalently led by the occluded perforating artery of MCA, its relevance to ICA calcification seems loose. Whether IAC has impact on territorial infarction requires further studies.

## Conclusions

The present multimodal imaging-based study revealed both similitude and difference between ICAD and IAC. Medial IAC is prevalently distributed along carotid siphon. Compared with plaques with medial IAC and non-calcified plaques, plaques with intimal IAC more frequently show luminal stenosis, eccentric plaque, larger plaque burden, and IPH, indicating a latent impact of intimal calcification on plaque vulnerability. Our findings may provide evidence for the clinical evaluation on mechanism, risk, and prognosis of ischemic stroke. The understanding of the mechanism in the presence of calcification will be deepened with further study upon the correlation of IAC and territorial infarctions.

## Data Availability Statement

The raw data supporting the conclusions of this article will be made available by the authors, without undue reservation.

## Ethics Statement

The studies involving human participants were reviewed and approved by the Clinical Research Ethics Committee of the Chinese University of Hong Kong. The patients/participants provided their written informed consent to participate in this study.

## Author Contributions

HD recruited patients, analyzed the imaging data, and drafted the manuscript. JL participated in the study design and recruited patients. WY acquired the vwMRI data and revised the manuscript. DB revised the manuscript. LZ analyzed the imaging data. LW and TL participated in the coordination of the study. XC conceived the study, participated in the study design and coordination, and revised the manuscript. All authors contributed to the article and approved the submitted version.

## Funding

This study has received funding by grants from the General Research Fund from Research Grants Council of Hong Kong (GRF, Project No. 14112916) and the Health and Medical Research Fund of Hong Kong (HMRF, Project No. 04152586).

## Conflict of Interest

The authors declare that the research was conducted in the absence of any commercial or financial relationships that could be construed as a potential conflict of interest.

## Publisher's Note

All claims expressed in this article are solely those of the authors and do not necessarily represent those of their affiliated organizations, or those of the publisher, the editors and the reviewers. Any product that may be evaluated in this article, or claim that may be made by its manufacturer, is not guaranteed or endorsed by the publisher.

## References

[1] Pikija S Magdič J Hojs-Fabjan T. Calcifications of vertebrobasilar arteries on CT: detailed distribution and relation to risk factors in 245 ischemic stroke patients. Biomed Res Int. (2013) 2013:918970. 10.1155/2013/918970 23984421 PMC3747337

[2] Yilmaz A Akpinar E Topcuoglu MA Arsava EM. Clinical and imaging features associated with intracranial internal carotid artery calcifications in patients with ischemic stroke. Neuroradiology. (2015) 57:501–6. 10.1007/s00234-015-1494-8 25633540

[3] Baek JH Yoo J Song D Kim YD Nam HS Heo JH. The Protective Effect of Middle Cerebral Artery Calcification on Symptomatic Middle Cerebral Artery Infarction. Stroke. (2017) 48:3138–41. 10.1161/STROKEAHA.117.017821 28939676

[4] Bos D Portegies ML van der Lugt A Bos MJ Koudstaal PJ Hofman A Krestin GP Franco OH Vernooij MW Ikram MA. Intracranial carotid artery atherosclerosis and the risk of stroke in whites: the Rotterdam Study. JAMA Neurol. (2014) 71:405–11. 10.1001/jamaneurol.2013.6223 24535643

[5] Woodcock RJ Jr Goldstein JH Kallmes DF Cloft HJ Phillips CD. Angiographic correlation of CT calcification in the carotid siphon. AJNR Am J Neuroradiol. (1999) 20:495–9. 10219418 PMC7056065

[6] de Weert TT Cakir H Rozie S Cretier S Meijering E Dippel DW. Intracranial internal carotid artery calcifications: association with vascular risk factors and ischemic cerebrovascular disease. AJNR Am J Neuroradiol. (2009) 30:177–84. 10.3174/ajnr.A1301 18842764 PMC7051707

[7] Wu XH Chen XY Fan YH Leung TW Wong KS. High extent of intracranial carotid artery calcification is associated with downstream microemboli in stroke patients. J Stroke Cerebrovasc Dis. (2017) 26:442–7. 10.1016/j.jstrokecerebrovasdis.2016.10.007 27818028

[8] Kockelkoren R Vos A Van Hecke W Vink A Bleys RL Verdoorn D. Computed tomographic distinction of intimal and medial calcification in the intracranial internal carotid artery. PLoS ONE. (2017) 12:e0168360. 10.1371/journal.pone.0168360 28060941 PMC5218397

[9] Vos A Van Hecke W Spliet WG Goldschmeding R Isgum I Kockelkoren R. Predominance of nonatherosclerotic internal elastic lamina calcification in the intracranial internal carotid artery. Stroke. (2016) 47:221–3. 10.1161/STROKEAHA.115.011196 26514193

[10] Yang WJ Zheng L Wu XH Huang ZQ Niu CB Zhao HL. Postmortem study exploring distribution and patterns of intracranial artery calcification. Stroke. (2018) 49:2767–9. 10.1161/STROKEAHA.118.022591 30355206

[11] Compagne KCJ Clephas PRD Majoie C Roos Y Berkhemer OA van Oostenbrugge RJ. Intracranial carotid artery calcification and effect of endovascular stroke treatment. Stroke. (2018) 49:2961–8. 10.1161/STROKEAHA.118.022400 30571406 PMC6257510

[12] Gocmen R Arsava EM Oguz KK Topcuoglu MA. Atherosclerotic intracranial internal carotid artery calcification and intravenous thrombolytic therapy for acute ischemic stroke. Atherosclerosis. (2018) 270:89–94. 10.1016/j.atherosclerosis.2018.01.035 29407893

[13] Li F McDermott MM Li D Carroll TJ Hippe DS Kramer CM. The association of lesion eccentricity with plaque morphology and components in the superficial femoral artery: a high-spatial-resolution, multi-contrast weighted CMR study. J Cardiovasc Magn Reson. (2010) 12:37. 10.1186/1532-429X-12-37 20591197 PMC2904754

[14] Yang WJ Abrigo J Soo YO Wong S Wong KS Leung TW. Regression of plaque enhancement within symptomatic middle cerebral artery atherosclerosis: a high-resolution MRI study. Front Neurol. (2020) 11:755. 10.3389/fneur.2020.00755 32849214 PMC7399098

[15] Chen XY Lam WW Ng HK Fan YH Wong KS. The frequency and determinants of calcification in intracranial arteries in Chinese patients who underwent computed tomography examinations. Cerebrovasc Dis. (2006) 21:91–7. 10.1159/000090206 16340183

[16] Bleeker L Marquering HA van den Berg R Nederkoorn PJ Majoie CB. Semi-automatic quantitative measurements of intracranial internal carotid artery stenosis and calcification using CT angiography. Neuroradiology. (2012) 54:919–27. 10.1007/s00234-011-0998-0 22205339 PMC3435515

[17] Stary HC. Natural history of calcium deposits in atherosclerosis progression and regression. Z Kardiol. (2000) 89:28–35. 10.1007/s003920070097 10769401

[18] Qureshi AI Caplan LR. Intracranial atherosclerosis. Lancet. (2014). 383:984–98. 10.1016/S0140-6736(13)61088-0 24007975

[19] Wang Y Zhao X Liu L Soo YO Pu Y Pan Y. Prevalence and outcomes of symptomatic intracranial large artery stenoses and occlusions in China: the Chinese Intracranial Atherosclerosis (CICAS) Study. Stroke. (2014) 45:663–9. 10.1161/STROKEAHA.113.003508 24481975

[20] Feng X Chan KL Lan L Abrigo J Liu J Fang H. Stroke mechanisms in symptomatic intracranial atherosclerotic disease: classification and clinical implications. Stroke. (2019) 50:2692–9. 10.1161/STROKEAHA.119.025732 31409268

[21] Ohara T Toyoda K Otsubo R Nagatsuka K Kubota Y Yasaka M. Eccentric stenosis of the carotid artery associated with ipsilateral cerebrovascular events. AJNR Am J Neuroradiol. (2008) 29:1200–3. 10.3174/ajnr.A0997 18339721 PMC8118845

[22] Leung TW Wang L Zou X Soo Y Pu Y Ip HL. Plaque morphology in acute symptomatic intracranial atherosclerotic disease. J Neurol Neurosurg Psychiatry. (2020) 92:370–6. 10.1136/jnnp-2020-325027 33239439 PMC7958085

[23] Mossa-Basha M Hwang WD De Havenon A Hippe D Balu N Becker KJ. Multicontrast high-resolution vessel wall magnetic resonance imaging and its value in differentiating intracranial vasculopathic processes. Stroke. (2015) 46:1567–73. 10.1161/STROKEAHA.115.009037 25953365

[24] Yang WJ Fisher M Zheng L Niu CB Paganini-Hill A Zhao HL. Histological characteristics of intracranial atherosclerosis in a Chinese population: a postmortem study. Front Neurol. (2017) 8:488. 10.3389/fneur.2017.00488 28993752 PMC5622314

[25] Wang Y Liu X Wu X Degnan AJ Malhotra A Zhu C. Culprit intracranial plaque without substantial stenosis in acute ischemic stroke on vessel wall MRI: A systematic review. Atherosclerosis. (2019) 287:112–21. 10.1016/j.atherosclerosis.2019.06.907 31254918 PMC6707846

[26] Lee HN Ryu CW Yun SJ. Vessel-wall magnetic resonance imaging of intracranial atherosclerotic plaque and ischemic stroke: a systematic review and meta-analysis. Front Neurol. (2018) 9:1032. 10.3389/fneur.2018.01032 30559708 PMC6287366

[27] Yang WJ Chen XY Zhao HL Niu CB Xu Y Wong KS. In vitro assessment of histology verified intracranial atherosclerotic disease by 15T magnetic resonance imaging: concentric or eccentric? Stroke. (2016) 47:527–30. 10.1161/STROKEAHA.115.011086 26628387

[28] Cao Y Sun Y Zhou B Zhao H Zhu Y Xu J. Atherosclerotic plaque burden of middle cerebral artery and extracranial carotid artery characterized by MRI in patients with acute ischemic stroke in China: association and clinical relevance. Neurol Res. (2017) 39:344–50. 10.1080/01616412.2017.1281196 28136710

[29] Ran Y Wang Y Zhu M Wu X Malhotra A Lei X. Higher plaque burden of middle cerebral artery is associated with recurrent ischemic stroke: a quantitative magnetic resonance imaging study. Stroke. (2020) 51:659–62. 10.1161/STROKEAHA.119.028405 31856694

[30] Virmani R Kolodgie FD Burke AP Finn AV Gold HK Tulenko TN. Atherosclerotic plaque progression and vulnerability to rupture: angiogenesis as a source of intraplaque hemorrhage. Arterioscler Thromb Vasc Biol. (2005) 25:2054–61. 10.1161/01.ATV.0000178991.71605.18 16037567

[31] Altaf N MacSweeney ST Gladman J Auer DP. Carotid intraplaque hemorrhage predicts recurrent symptoms in patients with high-grade carotid stenosis. Stroke. (2007) 38:1633–5. 10.1161/STROKEAHA.106.473066 17379827

[32] Xu WH Li ML Gao S Ni J Yao M Zhou LX. Middle cerebral artery intraplaque hemorrhage: prevalence and clinical relevance. Ann Neurol. (2012) 71:195–8. 10.1002/ana.22626 22367991

[33] Chen XY Wong KS Lam WW Zhao HL Ng HK. Middle cerebral artery atherosclerosis: histological comparison between plaques associated with and not associated with infarct in a postmortem study. Cerebrovasc Dis. (2008) 25:74–80. 10.1159/000111525 18033961

[34] Chen XY. and Fisher M. Pathological characteristics. Front Neurol Neurosci. (2016) 40:21–33. 10.1159/000448267 27960191 PMC6048595

[35] Nicoll R Henein MY. Arterial calcification: friend or foe? Int J Cardiol. (2013). 167:322–7. 10.1016/j.ijcard.2012.06.110 22809537

[36] Teng Z He J Degnan AJ Chen S Sadat U Bahaei NS. Critical mechanical conditions around neovessels in carotid atherosclerotic plaque may promote intraplaque hemorrhage. Atherosclerosis. (2012) 223:321–6. 10.1016/j.atherosclerosis.2012.06.015 22762729 PMC3437553

[37] Yang WJ Wong KS Chen XY. Intracranial atherosclerosis: from microscopy to high-resolution magnetic resonance imaging. J Stroke. (2017) 19:249–60. 10.5853/jos.2016.01956 28877564 PMC5647638

[38] Park KY Kim YB Moon HS Suh BC Chung PW. Association between cerebral arterial calcification and brachial-ankle pulse wave velocity in patients with acute ischemic stroke. Eur Neurol. (2009) 61:364–70. 10.1159/000210549 19365129

[39] Hernández-Pérez M Bos D Dorado L Pellikaan K Vernooij MW López-Cancio E N et al. Intracranial carotid artery calcification relates to recanalization and clinical outcome after mechanical thrombectomy. Stroke. (2017) 48:342–7. 10.1161/STROKEAHA.116.015166 28008095

